# Antimicrobial potential of probiotic cell‐free and *Carum copticum* L. seed extracts co‐nanoencapsulated in cellulose acetate fibers

**DOI:** 10.1002/fsn3.2893

**Published:** 2022-04-22

**Authors:** Maryam Azizkhani, Rafat Karbakhsh Ravari

**Affiliations:** ^1^ 600556 Department of Food Hygiene Faculty of Veterinary Medicine Amol University of Special Modern Technologies Amol Iran

**Keywords:** cellulose acetate, electrospinning, nanoencapsulation, probiotic, zenyan

## Abstract

The aim of this work was to co‐nanoencapsulate *Lactobacillus acidophilus* (LCFE) and *Bifidobacterium bifidum* (BCFE) cell‐free extract and zenyan (*Carum copticum* L.) seed water (ZWE) and ethanolic (ZEE) extract in electrospun cellulose acetate (CA) nanofibers and evaluate antimicrobial potential. The zeta potential, *SEM* image, antibacterial (MIC and MBC), and antifungal (MIC and MFC) activities were evaluated. TPC (total phenol content) of water and ethanol extract of zenyan seed were 14.05 and 136.44 mg GAE/g, respectively. A zeta potential of −40.25, −45.80, −43.71, 48.55, 35.50, 47.93, 31.50, 44.69, and −29.61 mV was found for nanofibers of pure CA (cellulose acetate), CA/LCFE, CA/BCFE, CA/ZWE, CA/ZEE, CA/LCFE/ZWE, CA/LCFE/ZEE, CA/BCFE/ZWE, and CA/LCFE/ZEE, respectively. CA electrospun nanofiber loaded with different extracts showed nanosized diameter and uniform structure. Nanoencapsulated extracts showed considerably higher antibacterial and antifungal activity compared to free extracts. Antibacterial activity of lactobacilli cell‐free extract was higher than bifidobacteria, which indicated the presence of the higher amount of antibacterial compounds in lactobacilli extract. Gram‐positive bacteria (*S. aureus* and *L. monocytogenes*) had the lowest MIC and MBC of free and nanoencapsulated extracts while Gram‐negatives (*E*. *coli*, *S. dysenteriae*, and *S. enteritidis*) had higher MIC and MBC. CA‐coated zenyan extracts (water and ethanolic) inhibited the growth of the assayed fungi at the MIC ranging 0.25 to 0.95%. These concentrations were 1.5–2 times lower than those obtained for pure extracts. For nanoencapsulated cell‐free extracts of both probiotics, the MIC values were about five times lower than the free extracts. The highest antimicrobial activity obtained for CA nanofibers contained zenyan ethanolic extract and cell‐free extract of lactobacilli or bifidobacteria.

## Graphical abstract

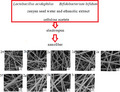

## 1 INTRODUCTION

The unregulated and extensive consumption of antibiotics to breed and treat livestock and poultry and treat human infections has led to the emergence of antibiotic‐resistant microorganisms and concerns about public health (Blair et al., 2015). In the recent decades, many studies have concentrated on the finding and production of natural antimicrobial compounds with high efficacy using new technologies (Liakos et al., 2017; Royo et al., 2010).

Among the antimicrobials of natural origin used in food and pharmaceutical industries, bacterial and plant metabolites are being popular. Probiotic bacteria, mainly bifidobacteria and lactobacilli, as the most used microorganisms in the human diet, produce multiple metabolites like bacteriocins, organic acids, enzymes, fatty acids, vitamins, proteins, peptides, etc. (Saadatzadeh et al., 2013). The species *Bifidobacterium animalis* colonizes in the mammalian colon and grows considerably in milk and milk‐derivate cultures. It shows resistance to low pH and oxidative stress, and its metabolites modulate the immune system and improve gut barrier function (Quigley, 2017). Also, it is reported that *B. bifidum* as a natural inhabitant of the human gut suppresses the gut inflammation and disturbance resulted from repeated antibiotic therapy (Ojima et al., 2020). The antimicrobial activity of some *Lactobacillus* species against *Enterobacteriaceae* and other pathogens is announced too (Chen et al., 2019; Inglin et al., 2015).

Phenolic compounds are mainly responsible for the antimicrobial and antioxidants activity of herbal extracts and essential oils. The extensive use of herbal extracts in the food industry to prevent lipid oxidation, retard microbial growth, delay food spoilage, and improve the organoleptic properties of food products has been reported (Parham et al., 2020). *Carrum copticum* L. (zenyan) of the family *Apiaceae* is cultivated in many parts of the world such as Iran and India. Traditionally, zenyan seed has been used as a flavoring agent and also for various therapeutic aspects such as respiratory distress, diarrhea, abdominal pain, and abdominal tumors. Several other health benefits including antibacterial, antifungal, and antiparasitic effects have been reported (Lim, 2012; Ramana et al., 2014). Zenyan seed contains important functional compounds like phenolics (carvacrol), thymol, terpinene, para‐cymene, and beta‐pinene (Alavinezhad & Boskabady, 2015; Mahmoudzadeh et al., 2017). The chemical compounds of plant extracts are volatile and easily degrade upon exposure to oxygen, high temperature, and light. Encapsulation and coating techniques such as micro‐ and nanoencapsulation, micro‐ and nanoemulsification, producing nanocomplexes, and micro‐ and nanofibers are used to deliver these sensitive compounds to their specific targets with the minimum loss and controlled release (Azizkhani et al., 2021a; Maes et al., 2019; Prakash et al., 2018; Wadhwa et al., 2017).

Electrospinning is a simple, easy applying, and novel technique to fabricate nanofibers that applies the electric force to draw charged threads of a polymer solution followed by exposure of the fibers to a spinning movement while transferring from a spinneret to a collector plate in the shape of ultrafine nonwoven fiber mats. This method allows producing nanomaterials with desired structural and physicochemical characteristics (Nagy et al., 2014; Xue et al., 2017; Zupančič et al., 2019). One of the biopolymers that can be easily electrospun to ultrafine fibers with nanometer size and possesses good biocompatibility, biodegradability, chemical resistance, and thermal stability is cellulose acetate (CA) (Liakos et al., 2015; Liakos et al., 2017). CA, a natural polymer, is the acetate ester of cellulose and the structural macromolecule of the green plants’ cell wall. The objective of the present research was to evaluate the antimicrobial activity of probiotics cell‐free and zenyan seed extracts co‐nanoencapsulated in electrospun CA nanofibers.

## 2 MATERIALS AND METHODS

### 2.1 Materials

All the chemicals, reagents, and culture media used in this work were purchased from Sigma‐Aldrich (USA) and Merck (Germany). Cellulose acetate (acetyl content: 38.7%; molecular weight: 29 kDa) was provided by Anmol Chemicals Co. (India).

### 2.2 Microbial strains

*Lactobacillus acidophilus* (LA5) and *Bifidobacterium bifidum* (both with human intestinal origin) were obtained from the Department of Microbiology, University of Turku (Turku, Finland). The bacterial pathogens *Escherichia coli* (ATCC 25922), *Staphylococcus aureus* (ATCC 29213), *Salmonella enteritidis* (ATCC 14028), *Listeria monocytogenes* (PTCC 1298), and *Shigella dysenteriae* (ATCC 13313) were obtained from the Faculty of Veterinary Science, University of Tehran (Tehran, Iran). *Aspergillus niger* (ATCC 9142), *Candida albicans (*ATCC 76615), *Fusarium* sp., and *Penicillium* sp. as the common fungi in food and feed contamination and spoilage were purchased from the Organization of Scientific and Industrial Research (Tehran, Iran).

### 2.3 Zenyan extract

Zenyan seeds were obtained from Medicinal Plants Research Institute (Karaj, Iran) and the verification of the genus and species was performed by the same institute. Ethanol and water extraction of zenyan was conducted according to Sun et al. method (Sun et al., 2015). Briefly, the seeds were ground to powder, mixed with water (100%) or 70% ethanol (ethanol: water 70:30), and stored on the shaker (150 rpm) (model 361, Fisher Scientific, USA) for 4 h. The extracts were kept in the dark bottles at 4ºC until used.

### 2.4 Measuring total phenolic content of zenyan extract

The total phenolic content (TPC) of zenyan water and ethanol extracts was determined using the Folin–Ciocalteu reagent (Şahin et al., 2013). The working solutions were prepared as follows: solution A contained 2% of aqueous Na_2_CO_3_ in 0.1 M NaOH; solution B contained 0.5% of aqueous CuSO_4_ in 1% NaKC_4_H_4_O_6_ solution; solution C was a mixture of solution A (50 ml) and solution B (1 ml) which was freshly prepared; and Folin–Ciocalteu reagent was made by diluting the stock solution with H_2_O at the ratio of 1:3 (v/v). To conduct the assay, 0.1 ml of the water or ethanol extract was added to 1.9 ml of H_2_O and 2.5 ml of solution C, and this mixture was incubated at ambient temperature for around 10 min. In the next step, 0.25 ml of the Folin–Ciocalteu reagent was added and kept at room temperature for 30 min in order to stabilize its blue color. The solutions’ absorbance was read by a spectrophotometer (model Lambda 365; Perkin Elmer, USA) at the wavelength of 750 nm. A standard calibration curve was plotted using multiple concentrations of gallic acid. The TPC was calculated from the standard curve and reported as mg of gallic acid equivalent (GAE) per g of the extract.

### 2.5 Preparation of probiotics cell‐free extract

*B. bifidum* were cultured in trypticase phytone yeast extract (TPY) broth and *L. acidophilus* in de Man Rogosa and Sharpe (MRS) broth and incubated at 37 ± 1°C for 48 h under anaerobic conditions (10% carbon dioxide, 10% hydrogen, and 80% nitrogen). Cell‐free extracts were obtained according to Burgut (2021). Briefly, the cells were harvested through four times centrifugation (model Z206A, Hermle, Germany) at 11,200 g, for 10 min at 4°C, rinsed twice, and resuspended in sterile deionized water followed by ultrasonic cell disruption (cell disruptor KS‐250F, Ningbo Haishu Kesheng Ultrasonic Equipments Co., China) (Burgut, 2021).

### 2.6 Preparing electrospun nanofibers

The nanoencapsulation process was carried out according to the method of Burgut (2021) with some modifications. Cell‐free extracts of *L. acidophilus* and *B. bifidum* were encapsulated individually. Co‐nanoencapsulation was performed with cell‐free extracts of bacteria, zenyan water, or ethanolic extract (1% v/v), and CA (10% w/v) were mixed. The mixtures were homogenized applying an ultrasonic homogenizer for 15 min (Sinosonics, China), and transferred into plastic syringes (attached to 23‐gauge stainless steel needles and a syringe pump), and electrospun by a high voltage power supply. The electrospinning process was carried out using a laboratory‐scale electrospinner (Vira System, Iran) and optimized to achieve desired nanofibers: different flow rates (1.0 to 6.0 ml/h), voltage values (70 to 150 kV), distances between the Taylor cone and the flat collector (8 to 22 cm), and the combinations of these parameters were tested. The optimization data are as follows: the voltage was adjusted at 125 kV, the flow rate of electrospinning dope solutions was 5 ml/h, and the distance between the needle and aluminum foil as the collector was adjusted at 15 cm. The electrospinning process was performed at 25 ± 1°C and the solutions were volatilized completely during the electrospinning. To remove the remained water, the obtained nanofibers were freeze dried after collecting from the aluminum foil. The average thickness of electrospun nanofiber mats was around 0.18 mm (Liakos et al., 2015; Liakos et al., 2017).

### 2.7 Zeta potential of nanofiber mats

The zeta potential is indicative of the stability of a colloidal dispersion and the electrophoretic mobility of the particles. The zeta potential of the electrospun nanofiber mats was measured applying the Zetasizer^®^ Nano ZS (model ZEN 3600, Malvern Instruments, Worcestershire, UK). The samples were prepared through the dispersion of 1 mg of nanofiber mats in 5 ml of PBS and run 10 times at 25 ± 1°C.

### 2.8 Scanning electronic microscopy (*SEM*)

To obtain *SEM* images, one layer of a two‐sided tape was attached to the sample place of the scanning electron microscope and the freshly fabricated samples of nanofiber mat were sprayed onto one side of the tape followed by gold spraying. The samples were observed on a high‐resolution and low‐vacuum scanning electron microscope (MIRA3 FEG‐*SEM*, Tescan Co., Czech).

### 2.9 Evaluation of antimicrobial activity

#### 2.9.1 Antibacterial activity

Antibacterial activity of nonencapsulated and nanoencapsulated probiotic and zenyan extracts was evaluated by determining the minimum inhibitory concentrations (MIC) and minimum bactericidal concentrations (MBC). The microdilution method in 96‐well microtiter plates was used to measure MIC and MBC according to Azizkhani et al. with some modifications (Azizkhani et al., 2021b). Briefly, the bacteria *E. coli*, *S. aureus*, *S. enteritidis*, *L. monocytogenes*, and *S. dysenteriae* were recovered in the BHI broth at 37 ± 1ºC for 18–24 h, and for each individual bacteria, the cell population was adjusted at 10^6^ CFU/ml. The nonencapsulated and nanoencapsulated probiotic and zenyan extracts were diluted, added to the BHI broth, transferred to the microwells up to 180 μl, and then 20 μl of the bacterial inoculum was added and shook horizontally. The microplates were incubated at 37 ± 1ºC for 24 h. MIC was determined as the lowest concentration in which no visible bacterial growth was observed. MBC was measured by a subculturing of 50 μl of the wells with no visible growth on the BHI agar after incubating at 37ºC.

#### 2.9.2 Antifungal activity

Antifungal activity tests were carried out according to the method described by Azizkhani et al. (2021a,b) and Kapustova et al. (2021) with modifications (Azizkhani et al., 2021b; Kapustová et al., 2021). The fungi (*C. albicans*, *A. niger*, *Penicillium* sp., and *Fusarium* sp.) were cultured on the slant potato dextrose agar (PDA) followed by incubation at 30 ± 1°C for 7 days. Then, 10 ml of sterile sodium lauryl sulfate (0.01% w/v in NaCl 1%) was transferred to the slant PDA to prepare monospore suspension. The obtained suspensions were passed through Whatman paper (pore size: 180 μm) and the fungal population was adjusted at 5 × 10^5^ conidia/ml. The antifungal experiment was performed on Petri dishes containing malt extract (1% w/v), yeast extract (2% w/v), and agar (2% w/v). After sterilizing and cooling to 45 ± 1ºC, the agar medium was mixed with the extracts (10% v/v) and transferred into the Petri dishes. The plates were inoculated by micropipetting of 10 μl of the conidia suspensions (5 × 10^5^ conidia/ml) in the center of the solidified culture medium. The diameter of the inoculums was considered as the initial diameter of the fungal colony. Inoculated Petri dishes were put in plastic boxes (containing bottles of water to prevent dehydration) and incubated at 25 ± 1ºC for 7 days. The diameter of the growth inhibition zone was calculated as:

$$\text{Growth inhibition zone}=D_{\text{control}}‐{\;D}_{\text{sample}}$$

where D_control_ was the mean diameter (mm) of the fungal colony in the control and D_sample_ was the mean diameter (mm) of the antifungal‐treated samples.

### 2.10 Data Analysis

Each experiment was conducted in triplicate and all the statistical analysis was performed by the software of SPSS 22.0 (SPSS Inc., Chicago, IL, USA), using the one‐way analysis of variance and the two‐sample *t*‐test. The significant differences were determined at the 95% and 99% levels.

## 3 RESULTS AND DISCUSSION

### 3.1 TPC of zenyan extracts

TPCs of water and ethanol extracts of zenyan 14.05 was 136.44 mg GAE/g of the extract, respectively. It is obvious from the results that ethanol extract had significantly higher TPC than water extract (*p* < .05). Higher TPC would result in higher antimicrobial and other functional activities. In a study by Khanavi et al. (2018), the TPC of ethanol extract of zenyan was 101.7–147.8 mg GAE/g depending on the extraction method (Khanavi et al., 2018). Zenyan mostly consists of monoterpenoids and polyphenols like flavonoids and phenolic acids (Zarshenas et al., 2013). In this study, the ethanolic extract had a higher polyphenols concentration compared to water extract. According to previous studies, the major compounds of water and ethanolic extracts of zenyan fruit are monoterpenoid glucosides like (2S, 6Z)‐3, 7‐dimethyloct 3(10)‐ene‐1, 2, 6, 7‐tetrol 1‐O‐β‐D‐glucopyranoside, and 6‐hydroxythymol 3‐O‐β‐D‐glucopyranoside; monoterpenoids, 3,7‐dimethyloct‐3(10)‐ene‐1, 2, 6, 7‐tetrol; glucosides such as 2‐methyl‐3‐buten‐2‐ol‐β‐D‐glucopyranoside benzyl‐β‐D‐glucopyranoside; and glucide like (3R)‐2‐hydroxymethylbutane1,2,3,4‐tetrol. Also, zenyan ethanolic extract mainly consists of thymol with great functional properties. The presence of compounds such as oxygenated carvacrol derivatives like thymol‐methyl‐ether and carvacrol‐methyl‐ether, *p*‐cymene, γ‐terpinene, α‐pinene, eucalyptol, and other minor components might have played an important role in improving the antimicrobial potential of the extracts (Mohagheghzadeh et al., 2007; Tooryan & Azizkhani, 2019; Zarshenas et al., 2013).

### 3.2 Zeta potential

**FIGURE 1:**
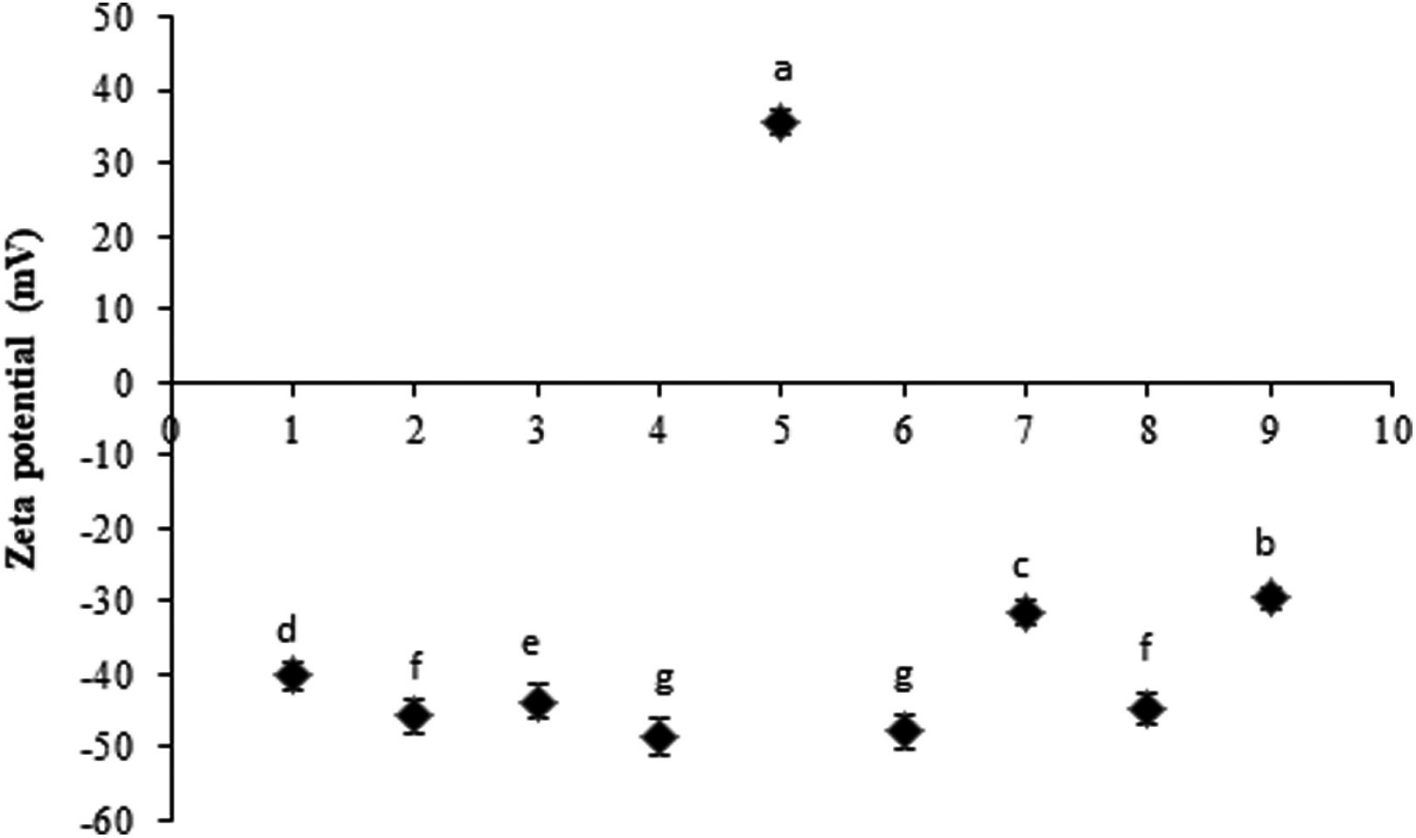
Zeta potential of CA nanofibers loaded with cell‐free extract of probiotics and zenyan extracts. CA: cellulose acetate; LCFE: *L. acidophilus* cell‐free extract; LCFE: *B. bifidum* cell‐free extract; ZWE: zenyan water extract; ZEE: zenyan ethanol extract

One of the most important and widely used characterization parameters for nanoscaled particles is zeta potential which is an indicator of the surface charge and the electrostatic potential. According to previous studies, well‐stabilized nanoparticles with high dispersion constancy showed a zeta potential value of ±30 mV which provides stable suspensions and prevents particle aggregation (Vogel et al., 2017). As presented in Figure 1, negative zeta potential values were found for all nanofibers except for CA loaded with zenyan ethanol extract (*p* < .05). It was found that the zeta potential of CA/extract nanofiber mats was lower (much negative) than the zeta potential value of pure CA nanofibers (*p* < .05). The variations in the zeta potential value were attributed to the presence of some compounds and molecules of the extracts on the outer surface of the nanofibers or nanoparticles (Liakos et al., 2018) and the changing manner of the zeta potential in our work shows that the extracts were grafted on the membrane of the CA biopolymer. The zeta potential of CA nanofibers loaded with zenyan ethanol extract was positive, while this value for the nanofibers containing zenyan water extract was found to be negative (*p* < .05). It is reported that herbal water and ethanol extracts present negative and positive zeta potentials, respectively, which is due to the surface charge of the extract components (Yuwono et al., 2015). Also, there was a slight difference between the zeta potential of the CA nanofibers loaded with cell‐free extracts of *L. acidophilus* and *B. bifidum* (*p* < .01).

Our data showed that loading CA nanofibers with probiotic cell‐free extracts increased the negativity of the zeta potential in comparison to control (without probiotics) which could be explained by the net negative charge and negative zeta potential value of the metabolites produced by *Lactobacillus* and *Bifidobacterium* (Dean et al., 2019; Ji et al., 2019; Murga et al., 2000; Pérez et al., 1998). The changes in the zeta potential value after loading nanofibers with the extracts expressed promising encapsulation of these extracts within CA biopolymer as a nanocarrier.

### 3.3 *SEM* images

**FIGURE 2:**
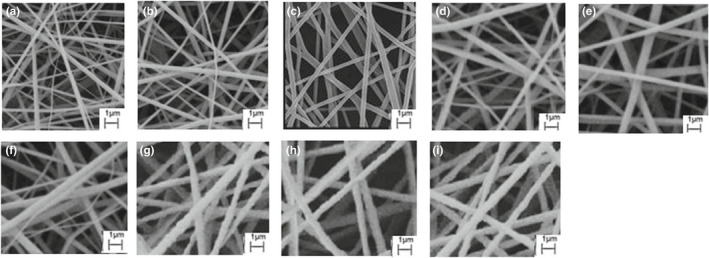
*SEM* image of (a): CA nanofibers; (b): CA nanofibers loaded with zenyan water extract; (c): CA nanofibers loaded with zenyan ethanol extract; (d): nanofibers loaded with cell‐free extract of *L. acidophilus*; (e): nanofibers loaded with cell‐free extract of *B. bifidum;* (f): nanofibers loaded with cell‐free extract of *L. acidophilus* and zenyan water extract; (g): nanofibers loaded with cell‐free extract of *L. acidophilus* and zenyan ethanol extract; (h): nanofibers loaded with cell‐free extract of *B. bifidum* and zenyan water extract; and (i): nanofibers loaded with cell‐free extract of *B. bifidum* and zenyan ethanol extract

**FIGURE 3:**
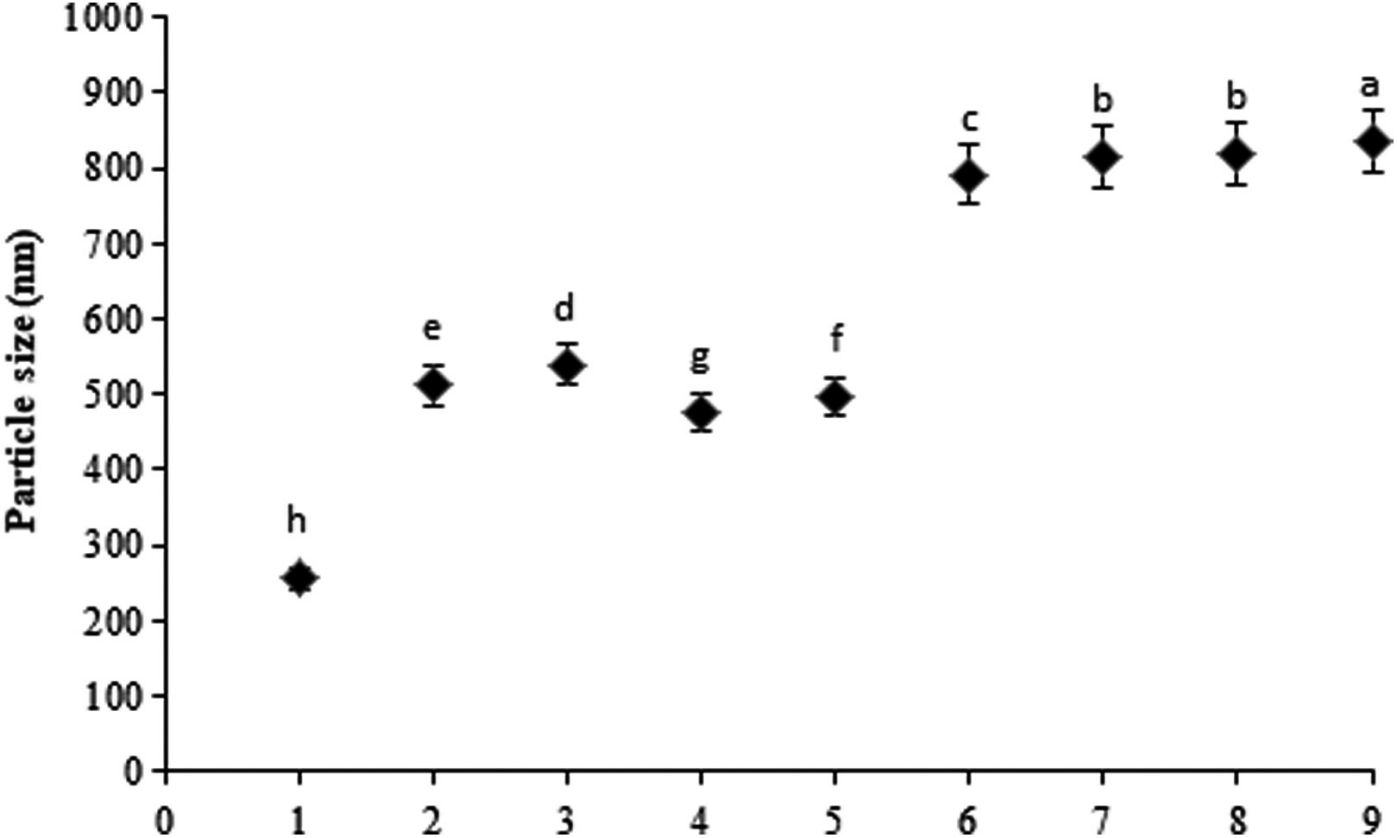
Diameter size of CA nanofibers loaded with cell‐free extract of probiotics and zenyan extracts. CA: cellulose acetate; LCFE: *L. acidophilus* cell‐free extract; LCFE: *B. bifidum* cell‐free extract; ZWE: zenyan water extract; ZEE: zenyan ethanol extract

The images of the control (CA nanofibers without extracts) and extract‐loaded electrospun nanofibers obtained by *SEM* are presented in Figure 2. The diameters of the nanofibers are calculated for 20 fibers for each image and ranged from 255 to 835 nm (Figure 3). According to the *SEM* data, CA nanofibers showed uniformity and fracture‐free or bead‐free morphology with an average diameter of around 285 nm. The CA fibers loaded with different extracts of zenyan and cell‐free extracts of bacteria had larger diameters than fibers of pure CA (*p* < .05). Also, extract‐loaded fibers showed less uniformity but continuous texture without considerable defect and fracture. From the *SEM* results, it was observed that loading CA with both zenyan extracts and cell‐free extracts of probiotics caused beads to appear in fibers and increased the diameter size (*p* < .05) but there was no fracture in fibers’ structure. In a study by Burgut (2021), the antimicrobial effect of co‐microencapsulated lactobacilli cell‐free and propolis ethanol and water extracts was evaluated. Wrinkled‐shaped microcapsules were found in capsules containing propolis water extracts (Burgut, 2021). In the current research, the absence of fractures and cracks in nanofibers revealed that the encapsulation process was carried out well.

The difference between the diameter size of extract‐loaded nanofibers is due to the chemical compounds of the extracts that as bioactive fractions react with hydroxyl moieties of CA and form hemiacetal bonds and uniform morphology or, in contrast, attached to CA as the host polymer and present less uniform and more complex fibers with higher diameters (Lammari et al., 2020).

### 3.4 Antimicrobial activity

#### 3.4.1 Antibacterial activity

The inhibitory activity of uncoated zenyan extract and cell‐free extracts of *L. acidophilus* and *B. bifidum* and their encapsulated form in CA nanofiber was evaluated against *E. coli*, *S. aureus*, *S. enteritidis*, *L. monocytogenes*, and *S. dysenteriae* (Table 1). It is apparent from the results that nanoencapsulated extracts showed significantly higher inhibitory effects on the growth of tested pathogens. Ethanol extract of zenyan had a stronger antigrowth capacity in comparison to water extract (*p* < .05). MIC and MBC of *B. bifidum* cell‐free extract were lower than *B. bifidum* that indicated the presence of the higher amount of antibacterial compounds in lactobacilli extract (*p* < .05). The combination of probiotics cell‐free extracts and zenyan extracts resulted in a considerable antibacterial activity, particularly upon nanoencapsulation (*p* < .05). Gram‐positive bacteria (*S. aureus* and *L. monocytogenes*) had the lowest MIC and MBC of free and nanoencapsulated extracts while Gram‐negative (*E*. *coli*, *S. dysenteriae*, and *S. enteritidis*) had higher MIC and MBCs (*p* < .05). Ethanolic and water extract of zenyan, encapsulated zenyan ethanol extract, and encapsulated cell‐free extract from probiotics with zenyan ethanolic and water extract had similar MIC and MBC against *L. monocytogenes*. However, microencapsulated extract from probiotics with zenyan ethanolic extract had lower MIC and MBC than that with zenyan water extract against other foodborne pathogens. Azmi et al. indicated that *Chromolaena odorata* extract encapsulated with methylcellulose and ethylcellulose had a stronger inhibitory effect on *S. aureus* and *E. coli* compared to free extract, which is in agreement with our results (Azmi et al., 2019).

**TABLE 1:** Antibacterial potential of probiotics cell‐free extracts and zenyan extracts and their encapsulated forms in CA nanofibers

	*Staphylococcus aureus*	*E. coli*	*Salmonella entritidis*	*Shigella dysenteriae*	*Listeria monocytogenes*
Free ZWE*					
MIC (%^†^)	2.00 ± 0.05^a^ ^‡^	2.50 ± 0.21^a^	2.45 ± 0.00^a^	2.40 ± 0.01^a^	2.10 ± 0.00^a^
MBC (%)	2.25 ± 0.15^b^	2.80 ± 0.13^b^	2.60 ± 0.00^a^	2.55 ± 0.01^a^	2.10 ± 0.05^a^
Free ZEE					
MIC (%)	1.25 ± 0.05^c^	1.80 ± 0.10^c^	1.60 ± 0.06^b^	1.65 ± 0.15^b^	1.35 ± 0.19^b^
MBC (%)	1.25 ± 0.09^c^	1.90 ± 0.23^c^	1.60 ± 0.02^b^	1.65 ± 0.20^b^	1.35 ± 0.11^b^
Free LCFE					
MIC (%)	3.10 ± 0.00^d^	3.60 ± 0.03^d^	3.50 ± 0.00^c^	3.50 ± 0.00^c^	3.15 ± 0.09^c^
MBC (%)	14.00 ± 0.00^e^	12.85 ± 0.02^e^	12.50 ± 0.00^c^	12.50 ± 0.01^c^	14.00 ± 0.05^d^
Free BCFE					
MIC (%)	3.85 ± 0.00^f^	4.75 ± 0.09^f^	4.70 ± 0.00^d^	4.65 ± 0.30^d^	4.05 ± 0.01^e^
MBC (%)	14.25 ± 0.009^g^	15.00 ± 0.14^g^	14.80 ± 0.05^e^	14.60 ± 0.09^d^	14.30 ± 0.20^f^
Free LCFE + ZWE					
MIC (%)	1.15 ± 0.00^c^	1.85 ± 0.02^c^	1.65 ± 0.03^b^	1.70 ± 0.08^b^	1.30 ± 0.00^b^
MBC (%)	3.50 ± 0.00^h^	4.90 ± 0.01^h^	4.50 ± 0.01^d^	4.45 ± 0.07^d^	3.85 ± 0.22^g^
Free BCFE + ZWE					
MIC (%)	1.40 ± 0.26^f^	2.15 ± 0.00^i^	2.00 ± 0.05^f^	2.00 ± 0.06^e^	1.55 ± 0.00^h^
MBC (%)	4.30 ± 0.05^i^	6.20 ± 0.05^j^	5.90 ± 0.00^g^	5.90 ± 0.07^f^	4.75 ± 0.09^i^
Free LCFE + ZEE					
MIC (%)	0.60 ± 0.00^j^	0.85 ± 0.01^k^	0.75 ± 0.00^h^	0.80 ± 0.03^g^	0.70 ± 0.00^j^
MBC (%)	1.10 ± 0.06^c^	1.70 ± 0.01^c^	1.55 ± 0.09^b^	1.50 ± 0.11^b^	1.35 ± 0.00^b^
Free BCFE + ZEE					
MIC (%)	0.65 ± 0.00^j^	1.05 ± 0.01^l^	0.95 ± 0.09^i^	0.95 ± 0.00^h^	0.80 ± 0.00^j^
MBC (%)	1.90 ± 0.05^k^	3.15 ± 0.20^m^	2.75 ± 0.33^a^	2.70 ± 0.00^a^	2.30 ± 0.00^k^
Encapsulated ZWE					
MIC (%)	0.65 ± 0.05^j^	0.95 ± 0.02^k^	0.90 ± 0.00^h^	0.90 ± 0.08^h^	0.75 ± 0.05^j^
MBC (%)	0.70 ± 0.05^j^	1.05 ± 0.05^l^	1.00 ± 0.01^h^	1.00 ± 0.06^h^	0.80 ± 0.10^j^
Encapsulated ZEE					
MIC (%)	0.40 ± 0.00^l^	0.65 ± 0.07^n^	0.55 ± 0.01^j^	0.55 ± 0.03^i^	0.45 ± 0.03^l^
MBC (%)	0.45 ± 0.05^l^	0.70 ± 0.01^n^	0.55 ± 0.09^j^	0.55 ± 0.01^i^	0.45 ± 0.05^l^
Encapsulated LCFE					
MIC (%)	2.80 ± 0.23^m^	3.20 ± 0.27^m^	3.00 ± 0.15^k^	3.05 ± 0.10^j^	2.90 ± 0.11^m^
MBC (%)	5.50 ± 0.16^m^	8.10 ± 0.42^o^	7.80 ± 0.60^l^	7.90 ± 0.51^k^	5.70 ± 0.62^n^
Encapsulated BCFE					
MIC (%)	2.90 ± 0.40^m^	4.05 ± 0.28^p^	4.00 ± 0.50^m^	4.00 ± 0.71^l^	3.00 ± 0.25^c^
MBC (%)	5.80 ± 0.09^m^	9.10 ± 0.14^q^	8.85 ± 0.35^n^	8.85 ± 0.19^l^	6.00 ± 0.50^o^
Encapsulated LCFE + ZWE					
MIC (%)	0.20 ± 0.00^o^	0.30 ± 0.02 u	0.25 ± 0.03^o^	0.25 ± 0.00^m^	0.20 ± 0.00^p^
MBC (%)	0.20 ± 0.06^o^	0.30 ± 0.05 u	0.25 ± 0.01^o^	0.25 ± 0.00^m^	0.20 ± 0.04^p^
Encapsulated BCFE + ZWE					
MIC (%)	0.20 ± 0.01^o^	0.35 ± 0.01 u	0.35 ± 0.00^p^	0.35 ± 0.03^n^	0.25 ± 0.00^p^
MBC (%)	0.20 ± 0.02^o^	0.40 ± 0.05 u	0.35 ± 0.04^p^	0.35 ± 0.01^n^	0.25 ± 0.05^p^
Encapsulated LCFE + ZEE					
MIC (%)	0.10 ± 0.00^p^	0.20 ± 0.02^x^	0.20 ± 0.00^o^	0.20 ± 0.01^m^	0.15 ± 0.00^q^
MBC (%)	0.10 ± 0.06^p^	0.20 ± 0.01^x^	0.20 ± 0.09^o^	0.20 ± 0.03^m^	0.15 ± 0.00^q^
Encapsulated BCFE + ZEE					
MIC (%)	0.10 ± 0.00^p^	0.20 ± 0.01^x^	0.20 ± 0.04^o^	0.20 ± 0.01^m^	0.15 ± 0.01^q^
MBC (%)	0.10 ± 0.02^p^	0.25 ± 0.00^x^	0.20 ± 0.00^o^	0.20 ± 0.00^m^	0.15 ± 0.01^q^

* ZWE: zenyan water extract; ZEE: zenyan ethanol extract; LCFE: *L. acidophilus* cell‐free extract; BCFE: *B. bifidum* cell‐free extract.

^†^ MIC and MBC are presented as % volume/volume.

^‡^ Different letters in the columns indicate statistically significant difference (*p* < .05). Data are presented as mean ± *SD*.

Several studies reported the antibacterial potential of micro‐ and nanoencapsulated extracts and essential oils within electrospun fibers against *E*. *coli*, *Yersinia enterocolitica*, *S. aureus*, *L. monocytogenes*, *S*. *typhimurium*, and *Bacillus cereus*, and demonstrated that Gram‐negative bacteria had higher MIC and MBC than Gram‐positive ones (Lammari et al., 2020; Liakos et al., 2017; Liakos et al., 2018). The most efficient nanofibers were the ones loaded with both cell‐free extracts of probiotics and zenyan ethanolic extract, which significantly inhibited the growth of bacterial pathogens. In a study by Burgut (2021), co‐microencapsulation of propolis extracts (mainly ethanolic extract) and cell‐free extract from *L*. *reuteri* led to greater inhibition zones against all the foodborne pathogen. The co‐microencapsulation of *L*. *reuteri* in combination with propolis water or ethanolic extract resulted in 2.2‐ and 2.34‐fold higher inhibition zone for *L. monocytogenes* (Burgut, 2021). This may be due to the combination of many bioactive ingredients possessing antimicrobial potential in zenyan extracts (Kazemi Oskuee et al., 2011). It is reported that cell‐free extract from lactobacilli consisted of the high amount of polyphenols, phenolic acids, organic acids, and alcohols that may play an important role in bacterial growth inhibition (Burgut, 2021; Chen et al., 2019).

#### 3.4.2 Antifungal activity

The antifungal effect of co‐encapsulated zenyan water and ethanolic extract with cell‐free extract of bifidobacteria and lactobacilli against some fungal food spoiling and human pathogens is presented in Table 2. The data showed marked antifungal activity of co‐encapsulated extracts in comparison to nonencapsulated extracts. The results revealed that CA‐coated zenyan extracts (water and ethanolic) inhibited the growth of the assayed fungi at the MIC values ranging from 0.25% to 0.95%. These concentrations were 1.5–2 times lower than those obtained for pure extracts (*p* < .05). For both nanoencapsulated probiotic cell‐free extracts, the MIC values were about five times lower than free extracts (*p* < .05). In particular, zenyan ethanolic extract had the minimum fungicidal concentration, among free extracts, against all tested fungi, while uncoated cell‐free extracts of bifidobacteria and lactobacilli showed high MFCs which revealed rather weak antifungal activity, especially against *C. albicans*. The empty CA nanofiber revealed no antifungal activity on the fungal growth (data not shown). As seen in Table 2, nanoencapsulation within CA fibers increased the antifungal effect significantly for all the herbal and probiotic cell‐free extracts (*p* < .05). MIC and MFC observed for the combination of free extract of zenyan and probiotics had no significant difference with these values for free water and ethanolic extracts of zenyan (*p* > .05). Nanoencapsulation considerably increased the antifungal activity of combined zenyan and probiotic cell‐free extracts (*p* < .05). Also, there was no significant difference between MIC and MFC of free and encapsulated extracts of *B. bifidum* and *L. acidophilus* (*p* > .05). *A. niger* and *C. albicans* were the most sensitive and resistant fungi upon antifungal treatments, respectively. In a study by Kapustova et al. (2021), the results evidenced that nanoencapsulated essential oils and extracts of *Origanum vulgare* and *Thymus capitatus* inhibited the growth of fungi at MIC values ranging from 0.12% to 0.25% (w/v) which were 2–4 times lower than values obtained for free (uncoated) extracts (Kapustová et al., 2021). It is reported that nanocapsules of oregano essential oil showed higher antifungal activity against *Penicillium* sp., *Fusarium* sp., and *Cladosporium* sp. compared to free essential oil (Bedoya‐Serna et al., 2018). Also, capsules of β‐cyclodextrin loaded with clove and oregano essential oils were recognized antifungal against *F. oxysporum* (Estrada‐Cano et al., 2017).

**TABLE 2:** Antifungal activity of probiotic cell‐free extracts and zenyan extracts and their encapsulated forms in CA nanofibers

	*Aspergillus niger*	*Penicillium* spp.	*Fusarium* spp.	*Candida albicans*
Free ZWE*				
MIC (%^*^)	1.00 ± 0.05^a,^ ^‡^	1.15 ± 0.09^a^	1.15 ± 0.08^a^	1.25 ± 0.07^a^
MFC (%)	2.00 ± 0.03^b^	2.30 ± 0.25^b^	2.30 ± 0.10^b^	2.50 ± 0.19^b^
Free ZEE				
MIC (%)	0.80 ± 0.00^c^	0.90 ± 0.01^c^	0.90 ± 0.01^c^	1.05 ± 0.07^c^
MFC (%)	1.60 ± 0.06^d^	1.80 ± 0.04^d^	1.80 ± 0.06^d^	2.10 ± 0.11^d^
Free LCFE				
MIC (%)	5.15 ± 0.86^e^	5.50 ± 0.74^e^	5.50 ± 0.42^e^	6.00 ± 0.53^e^
MFC (%)	10.30 ± 1.65^f^	11.00 ± 1.70^f^	11.00 ± 0.90^f^	12.00 ± 1.81^f^
Free BCFE				
MIC (%)	5.20 ± 0.00^e^	5.65 ± 0.09^e^	5.65 ± 0.00^g^	6.10 ± 0.30^e^
MFC (%)	10.40 ± 1.25^f^	11.30 ± 0.14^f^	11.30 ± 0.05^f^	12.20 ± 0.09^f^
Free LCFE + ZWE				
MIC (%)	0.95 ± 0.01^a^	1.10 ± 0.08^a^	1.10 ± 0.04^a^	1.30 ± 0.18^a^
MFC (%)	1.90 ± 0.23^b^	2.20 ± 0.30^b^	2.20 ± 0.25^b^	2.60 ± 0.05^b^
Free BCFE + ZWE				
MIC (%)	0.95 ± 0.05^a^	1.10 ± 0.02^a^	1.10 ± 0.05^a^	1.30 ± 0.07^a^
MFC (%)	1.90 ± 0.08^b^	2.20 ± 0.05^b^	2.20 ± 0.00^a^	2.60 ± 0.15^b^
Free LCFE + ZEE				
MIC (%)	0.70 ± 0.00^c^	0.90 ± 0.01^c^	0.90 ± 0.01^h^	0.95 ± 0.07^g^
MFC (%)	1.40 ± 0.09^g^	1.80 ± 0.02^d^	1.80 ± 0.05^b^	1.90 ± 0.31^h^
Free BCFE + ZEE				
MIC (%)	0.65 ± 0.03^c^	0.80 ± 0.01^g^	0.80 ± 0.09^g^	0.95 ± 0.00^g^
MFC (%)	1.20 ± 0.05^g^	1.60 ± 0.20^h^	1.60 ± 0.33^h^	1.90 ± 0.00^h^
Encapsulated ZWE				
MIC (%)	0.50 ± 0.04^h^	0.80 ± 0.13^g^	0.80 ± 0.05^g^	0.95 ± 0.01^g^
MFC (%)	1.00 ± 0.08^a^	1.60 ± 0.10^h^	1.60 ± 0.02^h^	1.90 ± 0.15^h^
Encapsulated ZEE				
MIC (%)	0.25 ± 0.00^i^	0.35 ± 0.01^i^	0.35 ± 0.00^i^	0.45 ± 0.04^i^
MFC (%)	0.50 ± 0.01^h^	0.70 ± 0.05^g^	0.70 ± 0.02^g^	0.90 ± 0.08^g^
Encapsulated LCFE				
MIC (%)	1.00 ± 0.05^a^	1.25 ± 0.27^i^	1.25 ± 0.28^j^	1.50 ± 0.20^j^
MFC (%)	2.00 ± 0.12^b^	2.50 ± 0.42^j^	2.50 ± 0.33^k^	3.00 ± 0.11^k^
Encapsulated BCFE				
MIC (%)	1.05 ± 0.09^a^	1.35 ± 0.28^i^	1.35 ± 0.30^j^	1.65 ± 0.07^l^
MFC (%)	2.10 ± 0.31^b^	2.70 ± 0.14^k^	2.70 ± 0.17^k^	3.30 ± 0.45^m^
Encapsulated LCFE + ZWE				
MIC (%)	0.10 ± 0.01^j^	0.20 ± 0.02^l^	0.20 ± 0.01^l^	0.25 ± 0.02^n^
MFC (%)	0.20 ± 0.03^k^	0.40 ± 0.05^m^	0.40 ± 0.07^m^	0.50 ± 0.01^i^
Encapsulated BCFE + ZWE				
MIC (%)	0.10 ± 0.00^j^	0.20 ± 0.01^l^	0.20 ± 0.00^l^	0.25 ± 0.04^n^
MFC (%)	0.20 ± 0.01^k^	0.40 ± 0.05^m^	0.40 ± 0.02^m^	0.50 ± 0.08^i^
Encapsulated LCFE + ZEE				
MIC (%)	0.05 ± 0.00^l^	0.10 ± 0.01^n^	0.10 ± 0.00^n^	0.15 ± 0.01^o^
MFC (%)	0.10 ± 0.06^j^	0.20 ± 0.01^l^	0.20 ± 0.06^l^	0.30 ± 0.02^n^
Encapsulated BCFE + ZEE				
MIC (%)	0.05 ± 0.00^l^	0.10 ± 0.01^n^	0.10 ± 0.00^n^	0.15 ± 0.02^o^
MFC (%)	0.10 ± 0.02^j^	0.20 ± 0.00^l^	0.20 ± 0.03^l^	0.30 ± 0.05^n^

* ZWE: zenyan water extract; ZEE: zenyan ethanol extract; LCFE: *L. acidophilus* cell‐free extract; BCFE: *B.bifidum* cell‐free extract.

^†^ MIC and MBC are presented as % volume/volume.

^‡^ Different letters in the columns indicate statistically significant difference (*p* < .05). Data are presented as mean ± *SD*.

These results are related to the chemical compounds of the extracts. As the main bioactive components of zenyan extracts, particularly ethanolic extract, consisted of polyphenols with high TPC value, their co‐encapsulation in CA fibers with cell‐free extracts of bifidobacteria and lactobacilli (rich in phenols; organic acids, e.g., acetic acid; hexadecanoic acid; octadecanoic acid; dihydroy benzoic acid; etc.) led to considerable antifungal activity (Burgut, 2021; Nazzaro et al., 2017).

## 4 CONCLUSION

The CA electrospun nanofibers loaded with water and ethanolic extracts of zenyan and cell‐free extracts of *L. acidophilus* and *B*. *bifidum* presented nanosized diameter and almost uniform and defect‐free structures. Also, they showed considerably higher antibacterial and antifungal activity compared to free extracts. The highest antimicrobial activity was found for CA nanofibers containing zenyan ethanolic extract and cell‐free extract of *L. acidophilus* and *B*. *bifidum*. The results of this work can be extended to food and pharmaceutical industrial experiments to introduce new generation of high‐efficiency preservatives. It is suggested that, in addition to industrial research, in vivo studies be conducted to investigate the clinical effects of this type of food additives.

## CONFLICT OF INTEREST

The authors declare that they do not have any conflict of interest.
